# Arachidonate lipoxygenases 5 is a novel prognostic biomarker and correlates with high tumor immune infiltration in low-grade glioma

**DOI:** 10.3389/fgene.2023.1027690

**Published:** 2023-01-27

**Authors:** Rui-han Pan, Xin Zhang, Zu-peng Chen, Ya-jun Liu

**Affiliations:** Department of Neurosurgery, The First Affiliated Hospital of Zhejiang Chinese Medical University, Hangzhou, Zhejiang, China

**Keywords:** arachidonate lipoxygenases 5 expression, methylation, immune cell infiltration, low-grade glioma, prognosis

## Abstract

**Objective:** To investigate the prognostic value of arachidonate lipoxygenases 5 (ALOX5) expression and methylation, and explore the immune functions of arachidonate lipoxygenases 5 expression in low-grade glioma (LGG).

**Materials and Methods:** Using efficient bioinformatics approaches, the differential expression of arachidonate lipoxygenases 5 and the association of its expression with clinicopathological characteristics were evaluated. Then, we analyzed the prognostic significance of arachidonate lipoxygenases 5 expression and its methylation level followed by immune cell infiltration analysis. The functional enrichment analysis was conducted to determine the possible regulatory pathways of arachidonate lipoxygenases 5 in low-grade glioma. Finally, the drug sensitivity analysis was performed to explore the correlation between arachidonate lipoxygenases 5 expression and chemotherapeutic drugs.

**Results:** arachidonate lipoxygenases 5 mRNA expression was increased in low-grade glioma and its expression had a notable relation with age and subtype (*p* < 0.05). The elevated mRNA level of arachidonate lipoxygenases 5 could independently predict the disease-specific survival (DSS), overall survival (OS), and progression-free interval (PFI) (*p* < 0.05). Besides, arachidonate lipoxygenases 5 expression was negatively correlated with its methylation level and the arachidonate lipoxygenases 5 hypomethylation led to a worse prognosis (*p* < 0.05). The arachidonate lipoxygenases 5 expression also showed a positive connection with immune cells, while low-grade glioma patients with higher immune cell infiltration had poor survival probability (*p* < 0.05). Further, arachidonate lipoxygenases 5 might be involved in immune- and inflammation-related pathways. Importantly, arachidonate lipoxygenases 5 expression was negatively related to drug sensitivity.

**Conclusion:** arachidonate lipoxygenases 5 might be a promising biomarker, and it probably occupies a vital role in immune cell infiltration in low-grade glioma.

## Introduction

Glioma is the most common primary malignant brain tumor characterized by a high disability rate, high recurrence rate, and high mortality rate (Wang et al., 2019). The molecular parameters and histology were incorporated into the 2016 version of the WHO classification of central nervous system tumors (Jiang T. et al., 2021). As of 2016, gliomas are divided into circumscribed gliomas (WHO grade I) and diffusely infiltrating gliomas (WHO grades II-IV) based on their pattern of growth and the IDH mutation status (Lapointe et al., 2018). Surgery, neuroimaging, chemotherapy, radiation therapy, and neuropathology are available approaches for the management of gliomas (Jiang et al., 2016). Circumscribed gliomas are benign and curable by complete surgical resection, while diffuse gliomas are almost never cured by resection alone (Louis et al., 2021). Histologically, grade II (low-grade) diffuse gliomas display nuclear atypia, grade III (anaplastic) show increased mitotic activity, and grade IV (glioblastomas) display additional microvascular proliferation, necrosis, or both (Lapointe et al., 2018). Diffuse low-grade gliomas (LGG) include grade II astrocytomas, oligodendrogliomas, and oligoastrocytomas (Jiang et al., 2016). After comprehensive treatments including surgical resection, chemotherapy, and radiotherapy, LGG patients have a better prognosis with a median survival of more than 10 years (Ding et al., 2022). However, they have inevitably suffered from recurrence and malignant progression due to the highly invasive nature of LGG (Zhao et al., 2022). Currently, biomarkers such as O6-methylguanine DNA methyltransferase (MGMT) and isocitrate dehydrogenase 1 (IDH1) have become vital markers of LGG clinical behavior and are closely connected with patient prognosis (Peng et al., 2022). Further development of prognostic indicators for LGG is urgently needed to gain additional insights to provide additional potential therapeutic targets.

Lipid peroxidation, which preferentially oxidizes polyunsaturated fatty acids, has been involved in the etiology of diverse pathological conditions such as diabetes and cancers (Anthonymuthu et al., 2016; Kuo et al., 2016; Zivkovic et al., 2017). Arachidonic acid (AA) is one of the most important polyunsaturated fatty acids in mammalian cells, which is required for normal cellular membrane fluidity and is the direct precursor of various bioactive mediators including endocannabinoids and leukotrienes (LTs) associated with inflammation (Zivkovic et al., 2017). AA is mainly metabolized by arachidonate lipoxygenases (ALOXs) which have six functional subtypes (Singh and Rao, 2019). ALOX5 is a non-heme iron-containing dioxygenase that encodes lipoxygenase and metabolizes AA into hydroperoxyderivatives (5-HPETE) and further into 5-hydroxyeicosatetraenoic acid (5-HETE), conferring growth, invasion, and chemopreventive advantage in cancer cells (Tang J. et al., 2021; Zhang et al., 2022). Kumar et al. demonstrated that the serum level of ALOX5 was elevated in breast cancer, and serves as a promising therapeutic target (Kumar et al., 2016). In addition, ALOX5 is upregulated in gastrointestinal cancer and its overexpression is related to the cancer progression (Merchant et al., 2018). However, the comprehensive role of ALOX5 in LGG remains unclarified.

In this study, we analyzed the expression level of ALOX5 in LGG and normal tissues followed by evaluating its prognostic value in LGG. Besides, the survival analysis of ALOX5 methylation and immune cell infiltration was conducted. We also performed functional enrichment analyses and revealed the molecular characteristic of ALOX5 in LGG. Finally, the drug sensitivity analysis was performed to explore the correlation between ALOX5 expression and sensitivity of chemotherapeutic drugs.

## Materials and methods

### Arachidonate lipoxygenases 5 gene expression analysis

The GEPIA database (http://gepia.cancer-pku.cn/) integrates TCGA big data for cancerous tissue and GTEx big data for normal tissue. This database was used to analyze the ALOX5 gene expression across all tumor samples and normal tissues, and to examine the expression of the ALOX5 gene in LGG and normal groups. Next, the immunohistochemistry (IHC) analysis was conducted to assess the ALOX5 protein level in LGG and normal tissues following the manufacturer’s protocol.

Then, normalized RNA-seq data and clinical information for LGG samples from the TCGA cohort were obtained from the UCSC Xena database (https://xenabrowser.net/), which has recomputed all raw expression data from TCGA. Inclusion criteria: 1) patients in WHO grades II gliomas; 2) with matched survival and expression data. Finally, 260 samples were enrolled in the study. The patients were divided into high- and low- ALOX5 expression groups with the median expression of ALOX5, and the association of ALOX5 with clinicopathological characteristics was analyzed using the chi-square test. Besides, we assessed the correlation between ALOX5 as a continuous variable and clinical parameters by *t*-test or ANOVA.

### Arachidonate lipoxygenases 5 gene survival analysis

Kaplan-Meier plotter curves were drawn to examine the correlation between ALOX5 mRNA expression and DFI, DSS, OS, and PFI of patients using the TCGA-LGG data. The log-rank test was used to assess the survival differences between high-and low- ALOX5 expression groups. Next, ROC analysis was conducted to evaluate the value of ALOX5 in predicting the survival status of LGG patients by using R package “timeROC” (Blanche et al., 2013), and the area under the curve (AUC) was calculated using the R package “pROC” (Robin et al., 2011). For validation, the glioma mRNA-seq dataset “mRNAseq_693” was acquired from the CGGA database (http://www.cgga.org.cn/). The Kaplan-Meier plotter method and ROC analysis were employed to verify the prognostic role of ALOX5 in LGG patients.

Following this, Cox regression analysis was performed to assess the independent prognostic factors for LGG patients using the TCGA and CGGA data. Nomogram models were constructed based on the multivariate Cox regression analysis results and calibration curves were drawn.

### Arachidonate lipoxygenases 5 methylation analysis

Gene Set Cancer Analysis (GSCA) (http://bioinfo.life.hust.edu.cn/GSCA/#/) is an integrated platform for genomic, pharmacogenomic, and immunogenomic gene set cancer analysis. We adopted this database to analyze the relationship between ALOX5 mRNA expression and its methylation level.

MEXPRESS (https://mexpress.be/) is a data visualization tool designed for the easy visualization of TCGA expression, DNA methylation, and clinical data, as well as the relationships between them. We downloaded the DNA methylation data of ALOX5 in LGG for analyzing its prognostic value.

### Immune cell infiltration analysis

To evaluate the correlation between ALOX5 expression and tumor immune microenvironment in LGG, the ESTIMATE algorithm was adopted to analyze the Immune score, Stromal score, and Estimate score. The effect of Immune score, Stromal score, and Estimate score on patient prognosis was explored using the Kaplan-Meier plotter method. Additionally, the association of ALOX5 expression with B cell, CD8 + T cell, CD4 + T cell, macrophage, neutrophil, and dendritic cell was evaluated using the TIMER algorithm. Similarly, the survival analyses of these immune cells were conducted by the Kaplan-Meier plotter method.

### Enrichment analysis

We used the R package “limma” to obtain differentially expressed genes (DEGs) between the high and low ALOX5 expression groups according to the median value of ALOX5 using the TCGA data (Yang et al., 2018). The significant DEGs were screened using the threshold of log 2 |foldchange (FC)| >1 and *p*-value < 0.05, and these DEGs were considered as LGG-related genes. Gene Ontology (GO) enrichment analysis and Kyoto Encyclopedia of Genes and Genomes (KEGG) pathway analysis of LGG-related genes were performed using the “clusterProfiler” in R (Yu et al., 2012).

Further, the gene set enrichment analysis (GSEA) was performed to illustrate the significant survival difference between the two expression groups. The gene set was permutated 1,000 times and the expression level of F12 was used as a phenotypic label. A nominal *p*-value <0.05 and an FDR q-value <0.25 were considered to be statistically significant.

Subsequently, the association of ALOX5 expression with immune activation-related genes and immune checkpoint-related genes in LGG was analyzed using the Pearson correlation test.

### Drug sensitivity analysis

We investigated the correlation between ALOX5 expression and drug response based on GDSC and CTRP in the GSCA database.

### Statistical analysis

All statistical analyses were performed in SPSS software (version 23.0) and packages of R (version 3.6.3). The *t*-test was used to analyze differences in each two-group comparison, and one-way ANOVA was employed to assess differences among at least three groups. Survival curves were drawn by the Kaplan-Meier method and differences in survival were compared by log-rank tests. *p* < 0.05 was considered statistically significant.

## Results

### Arachidonate lipoxygenases 5 gene expression analysis

**FIGURE 1:**
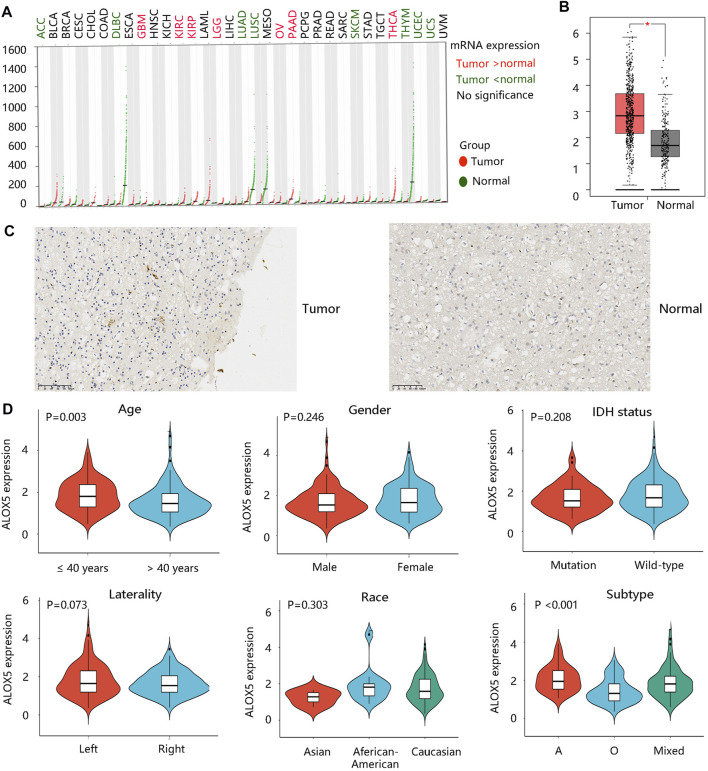
ALOX5 gene expression profile. **(A)** The ALOX5 gene expression in various cancers. **(B)** The higher ALOX5 expression in low grade glioma (LGG). **(C)** The protein level of ALOX5 in LGG and normal tissues by immunohistochemistry analysis. **(D)** The correlation between ALOX5 expression and clinicopathological characteristics.

Firstly, the ALOX5 gene expression in pan-cancer was explored through the GEPIA database as shown in Figure 1A. The ALOX5 mRNA level was significantly higher in the LGG group than that in the normal group (*p* < 0.01) (Figure 1B). Figure 1C showed the ALOX5 protein level in LGG and normal tissues. Besides, ALOX5 was significantly related to age and subtype (all *p* < 0.01), but had no significant relation with gender, IDH status, race, and laterality (Table 1) (Figure 1D). Of note, ALOX5 expression was upregulated in LGG patients aged ≤40 years compared with those aged >40 years. Astrocytoma patients seems to exhibit the highest ALOX5 mRNA expression, followed by the mixed type, and oligodendroglioma patients (all *p* < 0.01) (Figure 1D). These results suggested that high ALOX5 expression might be involved in the occurrence of LGG.

**TABLE 1:** Association of ALOX5 expression with clinicopathological characteristics in TCGA-LGG dataset.

Characteristics	Low ALOX5	High ALOX5	*p*-value
**Age**			0.001
≤40	63 (48.5%)	89 (68.5%)	
>40	67 (51.5%)	41 (31.5%)	
**Gender**			0.619
Male	63 (48.5%)	59 (45.4%)	
Female	67 (51.5%)	71 (54.6%)	
**Subtype**			<0.001
Astrocytoma	18 (13.8%)	47 (36.2%)	
Oligodendroglioma	78 (60.0%)	39 (30.0)	
Mixed	34 (26.2)	44 (33.8%)	
**IDH status**			0.385
Wild type	72 (69.9%)	82 (75.2%)	
Mutation	31 (30.1%)	27 (24.8%)	
**Race**			0.571
Asian	3 (2.4%)	1 (0.8%)	
African-American	6 (4.7%)	7 (5.6%)	
Caucasian	118 (92.9%)	118 (93.7%)	
**Laterality**			0.380
Left	61 (48.0%)	68 (53.5%)	
Right	66 (52.0%)	59 (46.5%)	

### High arachidonate lipoxygenases 5 gene expression predicted poor prognosis

**FIGURE 2:**
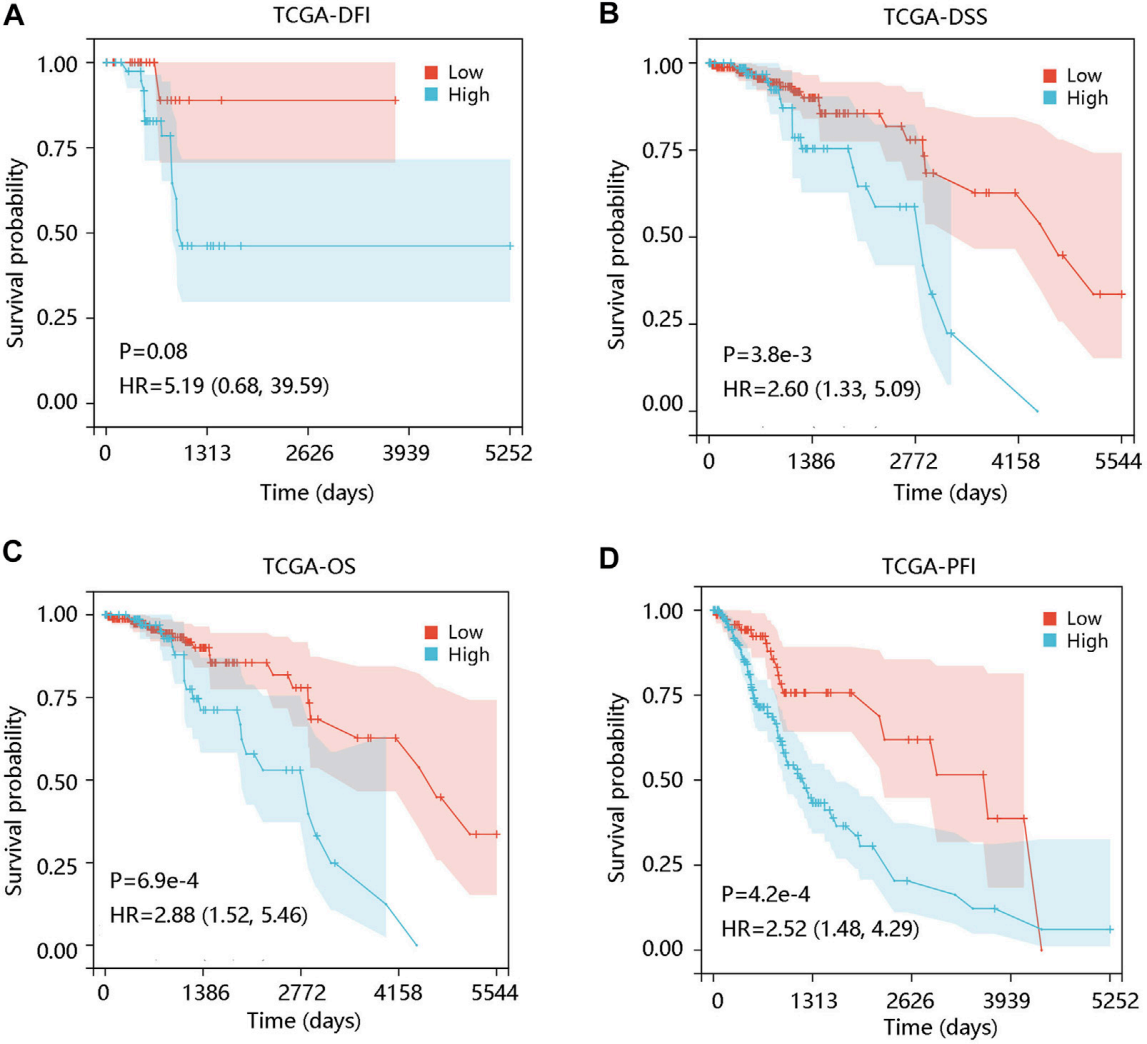
The association of ALOX5 expression with prognosis in low-grade glioma using the TCGA-LGG data. **(A)** High ALOX5 expression had no remarkable effect on DFI. High ALOX5 expression led to worse **(B)** DSS, **(C)** OS, and **(D)** PFI. DFI, disease-free interval; OS, overall survival; PFI, progression-free interval; HR, hazard ratio.

**FIGURE 3:**
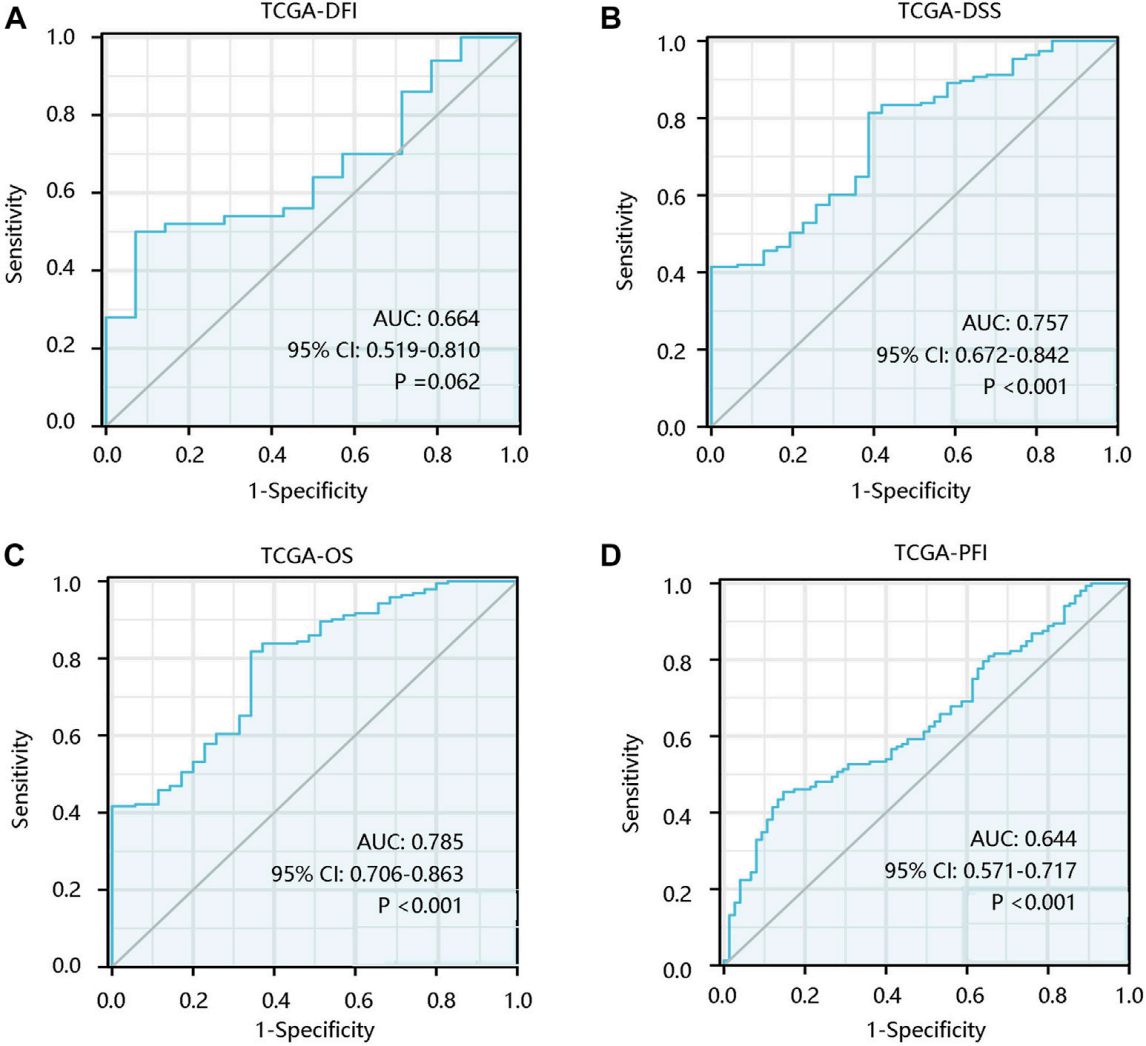
The predictive value of ALOX5 in low-grade glioma based on the TCGA-LGG data. The significance of ALOX5 in distinguishing the **(A)** DFI, **(B)** DSS, **(C)** OS, and **(D)** PFI statuses. DFI, disease-free interval; DSS, disease-specific survival; OS, overall survival; PFI, progression-free interval. AUC, area under the curve; 95% CI, 95% confidence interval.

To investigate the prognostic significance of ALOX5 gene in LGG, the survival curves were drawn using Kaplan-Meier plotter method based on TCGA-LGG data. ALOX5 expression was not significantly related to the DFI (*p* > 0.05) (Figure 2A), but patients with higher ALOX5 expression had shorter DSS, OS, and PFI with statistical significance (all *p* < 0.01) (Figures 2B–D). Although ALOX5 could not predict the DFI status well (AUC: 0.664; *p* > 0.05), it exhibited acceptable performance in predicting the statuses of DSS, OS, and PFI with AUCs of 0.757, 0.785, and 0.644, respectively (all *p* < 0.001) (Figures 3A–D).

**FIGURE 4:**
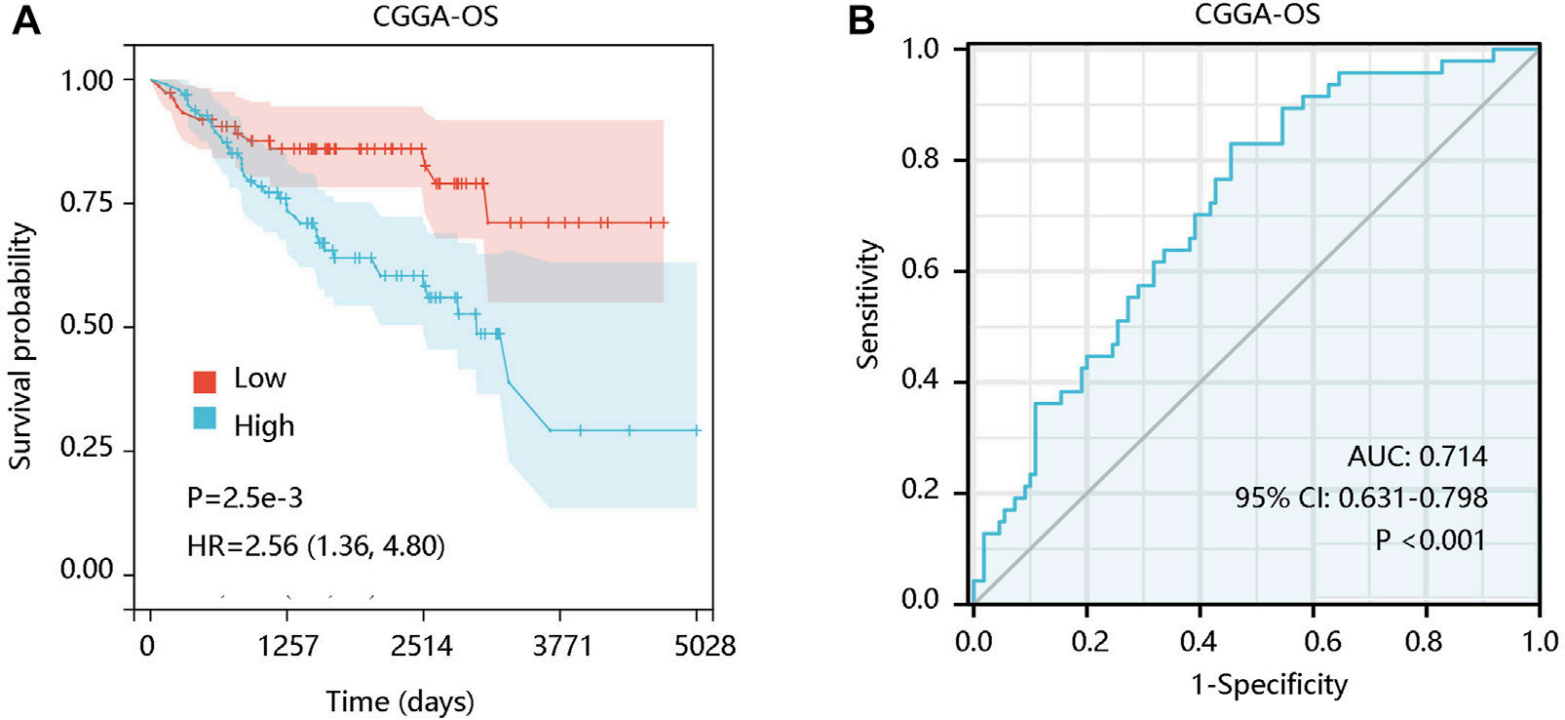
The prognostic value of ALOX5 gene in CGGA-LGG data. **(A)** Patients with high ALOX5 gene expression had poor OS. **(B)** ALOX5 expression presented satisfactory performance in predicting the OS status. OS, overall survival; AUC, area under the curve; 95% CI, 95% confidence interval; HR, hazard ratio.

For verification, the survival analysis was conducted using CGGA-LGG data. Expectedly, the increased ALOX5 expression contributed to unfavorable OS (*p* < 0.01) (Figure 4A), and its expression had the ability in distinguishing the OS status of LGG patients (AUC: 0.714; *p* < 0.001) (Figure 4B).

**FIGURE 5:**
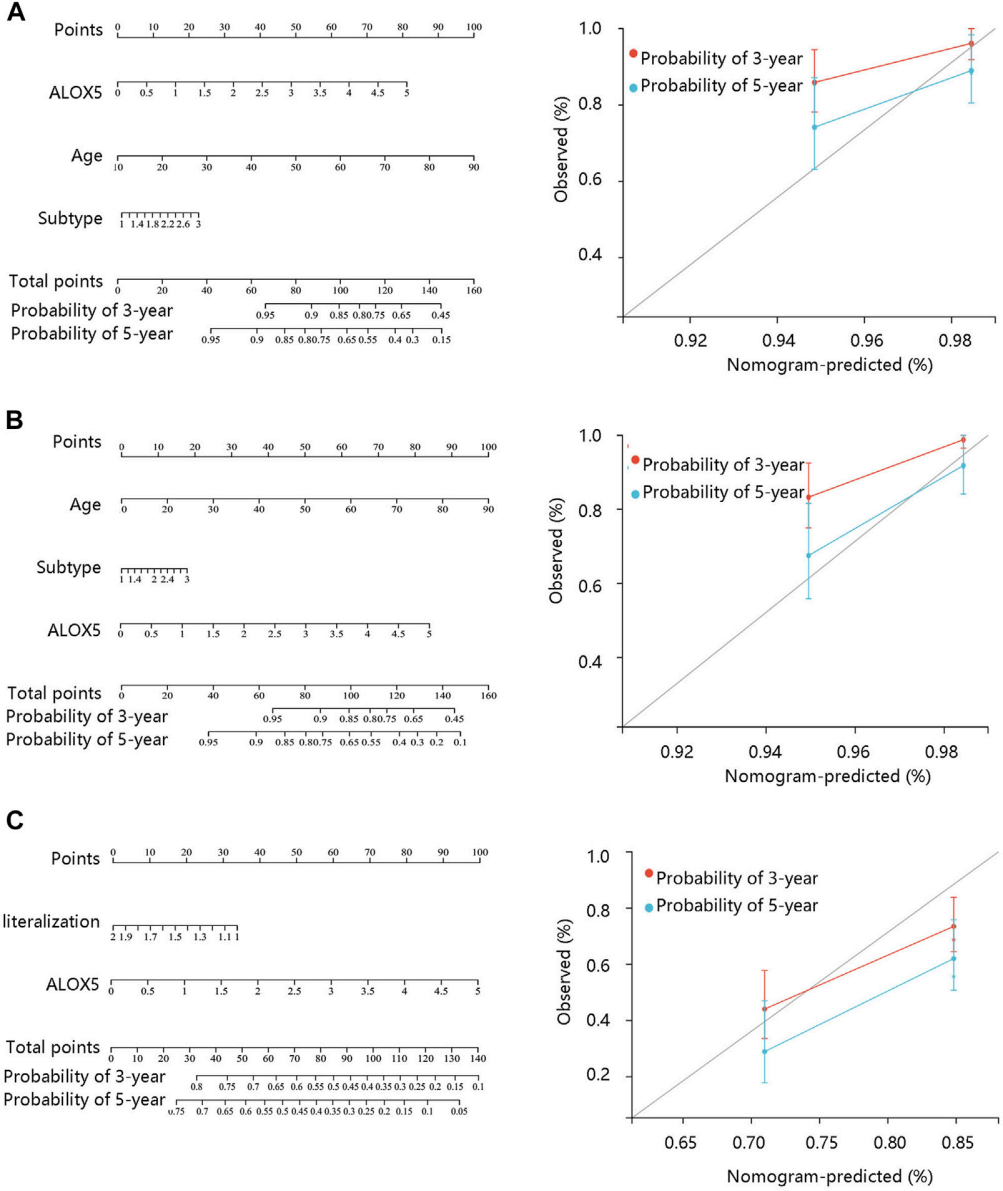
Nomogram construction and evaluation based on the multivariate Cox regression analysis results using the TCGA-LGG data. Nomogram was generated in terms of **(A)** disease-specific survival; **(B)** overall survival, and **(C)** progression-free interval.

**FIGURE 6:**
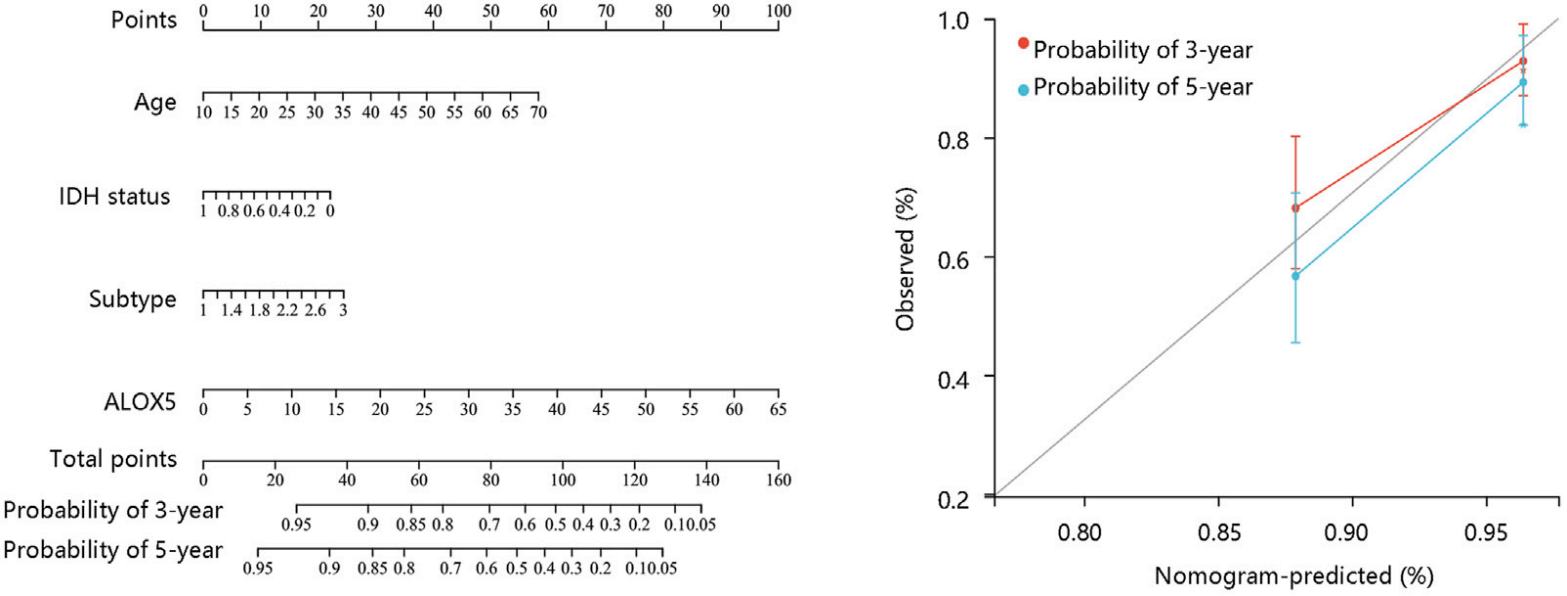
Nomogram construction and evaluation based on the multivariate Cox regression analysis results using the CGGA-LGG data.

To determine the independent prognostic role of ALOX5 in LGG, univariate and multivariate Cox regression analyses were performed using the TCGA-LGG data. The age, subtype, and ALOX5 were independent predictors for DSS and OS (all *p* < 0.05) (Table 2; Table 3; Table 4); while literality and ALOX5 were significantly related to PFI in the multivariate analysis (all *p* < 0.05) (Table 4). These independent factors were enrolled into the construction of nomogram models to predict the probability of DSS, OS, and PFI in LGG. The total number of points was calculated from the sum of the points assigned to each variable in the line graph. The calibration curves demonstrated an agreement between actual observations and predictions of the nomogram in the TCGA-LGG samples (Figures 5A–C). For validation, the Cox regression analysis was conducted using the CGGA-LGG data. The age, IDH status, subtype, and ALOX5 were independent predictors for OS (all *p* < 0.05) (Table 5). Among these independent factors, ALOX5 contributed the most to predicting the probability of OS in patients with LGG. The calibration curves also showed an agreement between actual observations and predictions of the nomogram in the CGGA-LGG samples (Figure 6). The above findings indicated that ALOX5 gene was significantly related to the LGG progression.

**TABLE 2:** Cox regression analysis of the association of ALOX5 and clinical variables with DSS in the TCGA-LGG dataset.

Characteristics (References)	Univariate analysis		Multivariate analysis	
	HR (95% CI)	*p*-Value	HR (95% CI)	*p*-Value
Age	1.04 (1.01–1.07)	0.005	1.05 (1.02–1.08)	0.001
Gender (female)	0.93 (0.49–1.76)	0.812	0.82 (0.38–1.78)	0.617
IDH status (wild type)	1.92 (0.84–4.38)	0.122	1.39 (0.55–3.52)	0.486
Race (Asian)	1.08 (0.30–3.83)	0.909	1.03 (0.26–4.16)	0.964
Subtype (astrocytoma)	1.37 (0.85–2.22)	0.196	1.85 (1.06–3.23)	0.032
Laterality (left)	0.56 (0.29–1.01)	0.087	0.49 (0.22–1.10)	0.085
ALOX5	1.53 (1.03–2.28)	0.034	1.59 (1.02–2.47)	0.039

Abbreviations: DSS, disease-specific survival; HR, hazard ratio; 95% CI, 95% confidence interval.

**TABLE 3:** Cox regression analysis of the association of ALOX5 and clinical variables with OS in the TCGA-LGG dataset.

Characteristics (References)	Univariate analysis		Multivariate analysis	
	HR (95% CI)	*p*-Value	HR (95% CI)	*p*-Value
Age	1.04 (1.01–1.06)	0.005	1.05 (1.02–1.08)	0.001
Gender (female)	0.96 (0.52–1.76)	0.891	0.83 (0.39–1.74)	0.615
IDH status (wild type)	1.80 (0.80–4.07)	0.157	1.32 (0.53–3.28)	0.557
Race (Asian)	1.11 (0.31–3.95)	0.871	1.08 (0.27–4.26)	0.914
Subtype (astrocytoma)	1.37 (0.87–2.15)	0.174	1.90 (1.10–3.25)	0.021
Laterality (left)	0.58 (0.31–1.10)	0.096	0.51 (0.23–1.10)	0.807
ALOX5	1.62 (1.12–2.35)	0.011	1.66 (1.09–2.53)	0.018

Abbreviations: OS, overall survival; HR, hazard ratio; 95% CI, 95% confidence interval.

**TABLE 4:** Cox regression analysis of the association of ALOX5 and clinical variables with PFI in the TCGA-LGG dataset.

Characteristics (References)	Univariate analysis		Multivariate analysis	
	HR (95% CI)	*p*-Value	HR (95% CI)	*p*-Value
Age	1.01 (0.99–1.02)	0.699	1.01 (0.98–1.02)	0.820
Gender (female)	0.88 (0.58–1.33)	0.538	0.71 (0.46–1.12)	0.142
IDH status (wild type)	1.05 (0.60–1.83)	0.874	0.52 (0.32–0.84)	0.731
Race (Asian)	0.89 (0.44–1.81)	0.747	0.72 (0.32–1.63)	0.431
Subtype (astrocytoma)	0.94 (0.71–1.25)	0.674	0.89 (0.66–1.19)	0.416
Laterality (left)	0.50 (0.32–0.78)	0.002	0.52 (0.32–0.84)	0.007
ALOX5	1.55 (1.20–1.99)	0.001	1.55 (1.18–2.05)	0.002

Abbreviations: PFI, progression-free interval; HR, hazard ratio; 95% CI, 95% confidence interval.

**TABLE 5:** Cox regression analysis of the association of ALOX5 and clinical variables with OS in the CGGA-LGG dataset.

Characteristics (References)	Univariate analysis		Multivariate analysis	
	HR (95% CI)	*p*-value	HR (95% CI)	*p*-value
Age	1.03 (0.99–1.06)	0.096	1.04 (1.01–1.07)	0.018
Gender (female)	1.03 (0.57–1.87)	0.929	1.15 (0.61–2.17)	0.665
IDH status (wild type)	0.44 (0.23–0.85)	0.015	0.44 (0.21–0.89)	0.023
Subtype (astrocytoma)	1.46 (1.02–2.09)	0.040	1.56 (1.10–2.22)	0.013
ALOX5	1.06 (1.03–1.09)	<0.001	1.06 (1.03–1.09)	<0.001

Abbreviations: OS, overall survival; HR, hazard ratio; 95% CI, 95% confidence interval.

### High methylation level of arachidonate lipoxygenases 5 was related to favorable prognosis

**FIGURE 7:**
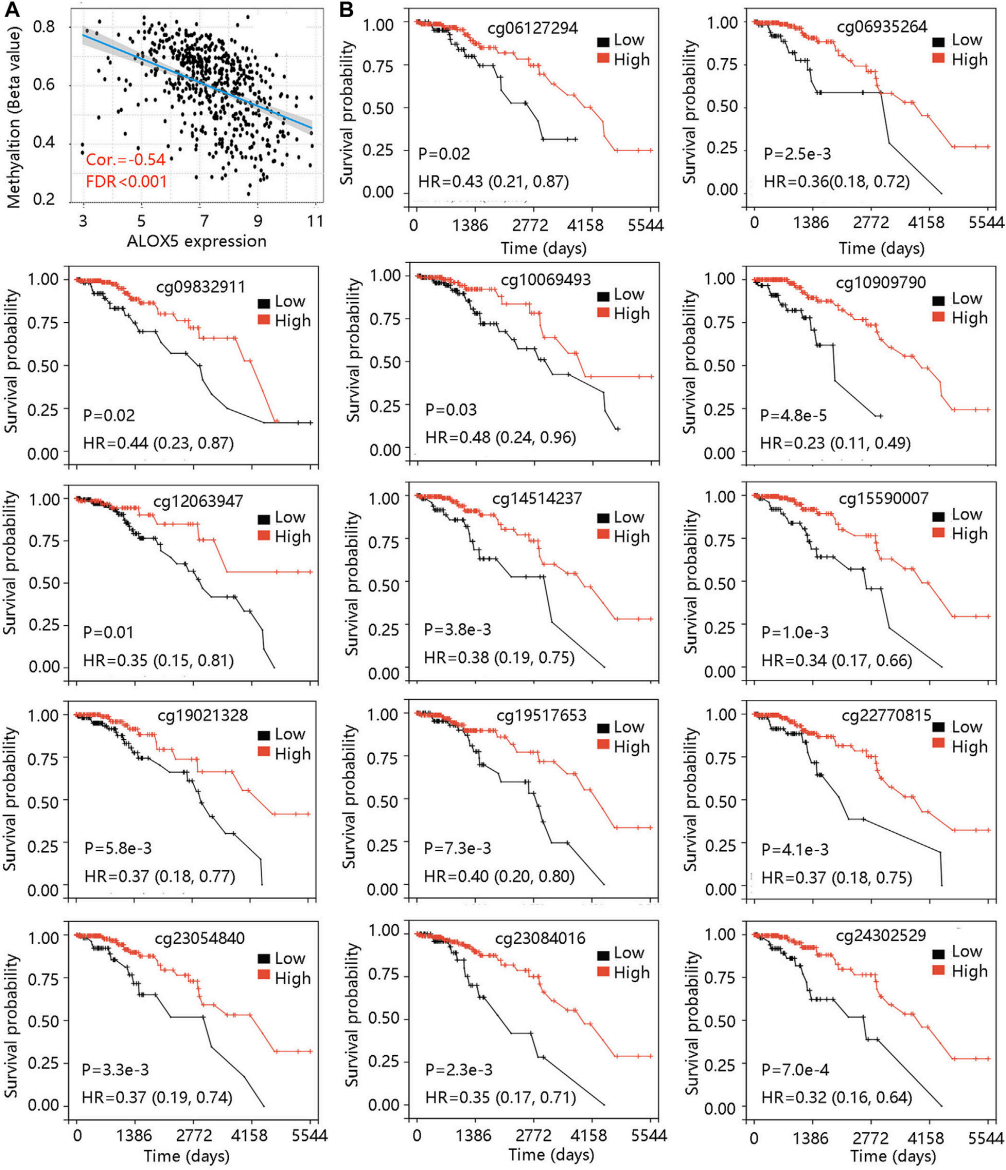
The ALOX5 methylation analysis in TCGA-LGG cohort. **(A)** The negative correlation between ALOX5 mRNA expression and its methylation level. **(B)** The prognostic value of the 14 methylated sites of ALOX5. FDR, false discover rate; HR, hazard ratio.

DNA methylation is an epigenetic alteration that plays an essential role in the development of several cancers (de Almeida et al., 2019). Pearson correlation test unveiled the negative correlation between ALOX5 mRNA expression and its methylation level (Figure 7A). Using the DNA methylation data in the TCGA-LGG cohort, we found that low methylation level groups of cg06127294, cg06935264, cg09832911, cg10069493, cg10909790, cg12063947, cg14514237, cg15590007, cg19021,328, cg19517653, cg22770815, cg23054840, cg23084016, and cg24302529 led to worse OS than their related high methylation level groups (all *p* < 0.05) (Figure 7B) (Table 6). The result suggested that the low methylation level of ALOX5 might affect its gene expression, and lead to unfavorable clinical outcomes for LGG patients.

**TABLE 6:** Summary of the Kaplan-Meier curve data for the 14 methylated sites of ALOX5 in the TCGA-LGG dataset.

Methylated sites	Hazard ratio	95% CI-low	95% CI-high	*p*-Value
cg06127294	0.43	0.21	0.87	0.020
cg06935264	0.36	0.18	0.72	0.003
cg09832911	0.44	0.23	0.87	0.020
cg10069493	0.48	0.24	0.96	0.030
cg10909790	0.23	0.11	0.49	<0.001
cg12063947	0.35	0.15	0.81	0.010
cg14514237	0.38	0.19	0.75	0.004
cg15590007	0.34	0.17	0.66	0.001
cg19021328	0.37	0.18	0.77	0.006
cg19517653	0.40	0.20	0.80	0.007
cg22770815	0.37	0.18	0.75	0.004
cg23054840	0.37	0.19	0.74	0.003
cg23084016	0.35	0.17	0.71	0.002
cg24302529	0.32	0.16	0.64	<0.001

Abbreviations: 95% CI, 95% confidence interval.

### High immune cell infiltration level was correlated with poor prognosis

**FIGURE 8:**
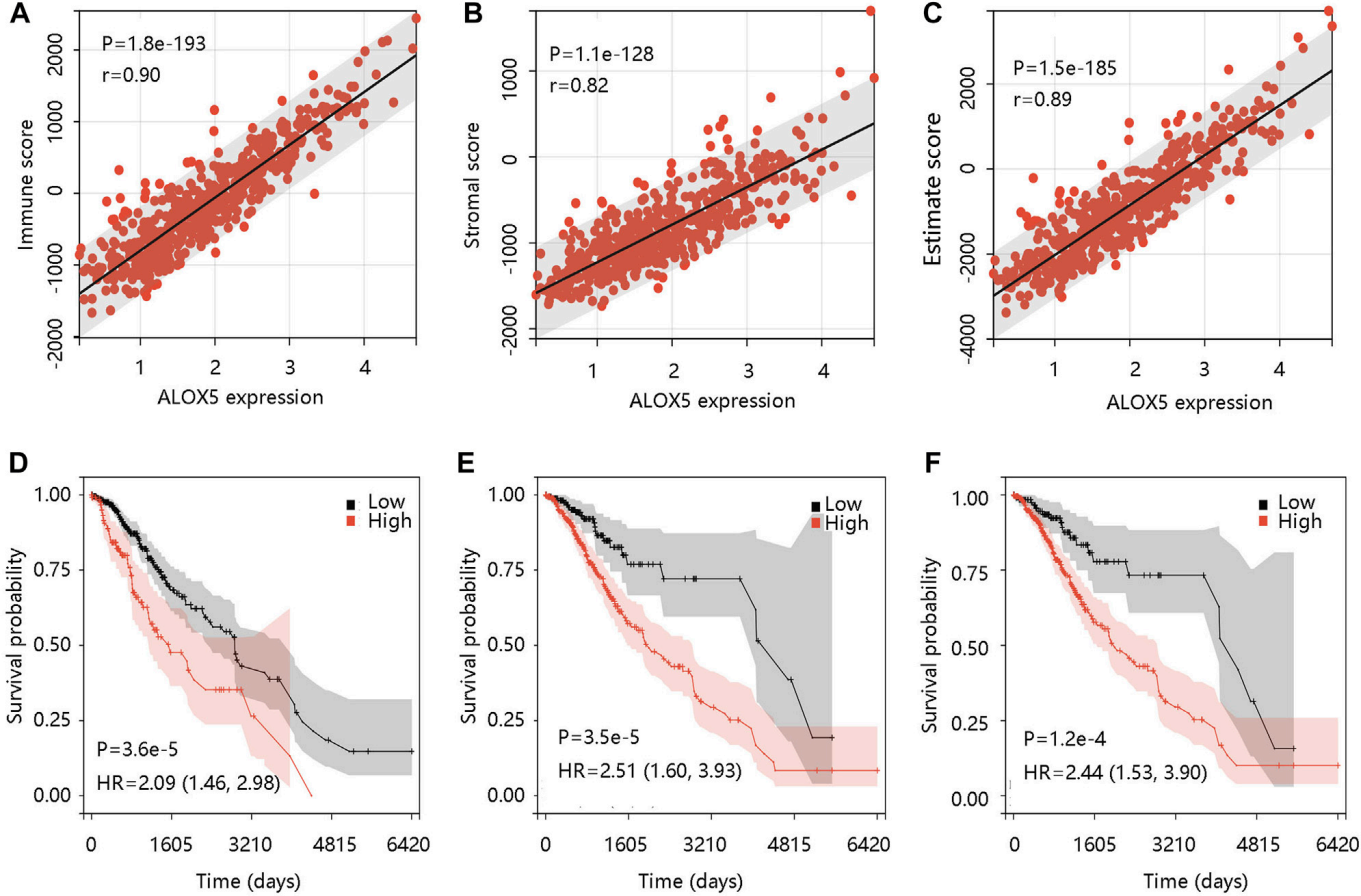
The immune cell infiltration analysis using ESTIMAE algorithm in TCGA-LGG data. The positive association of ALOX5 expression with **(A)** Immune score, **(B)** Stromal score, and **(C)** Estimate score. The correlation between the **(D)** Immune score-, **(E)** Stromal score-, and **(F)** Estimate score-based groups, and sample survival. HR, hazard ratio.

**FIGURE 9:**
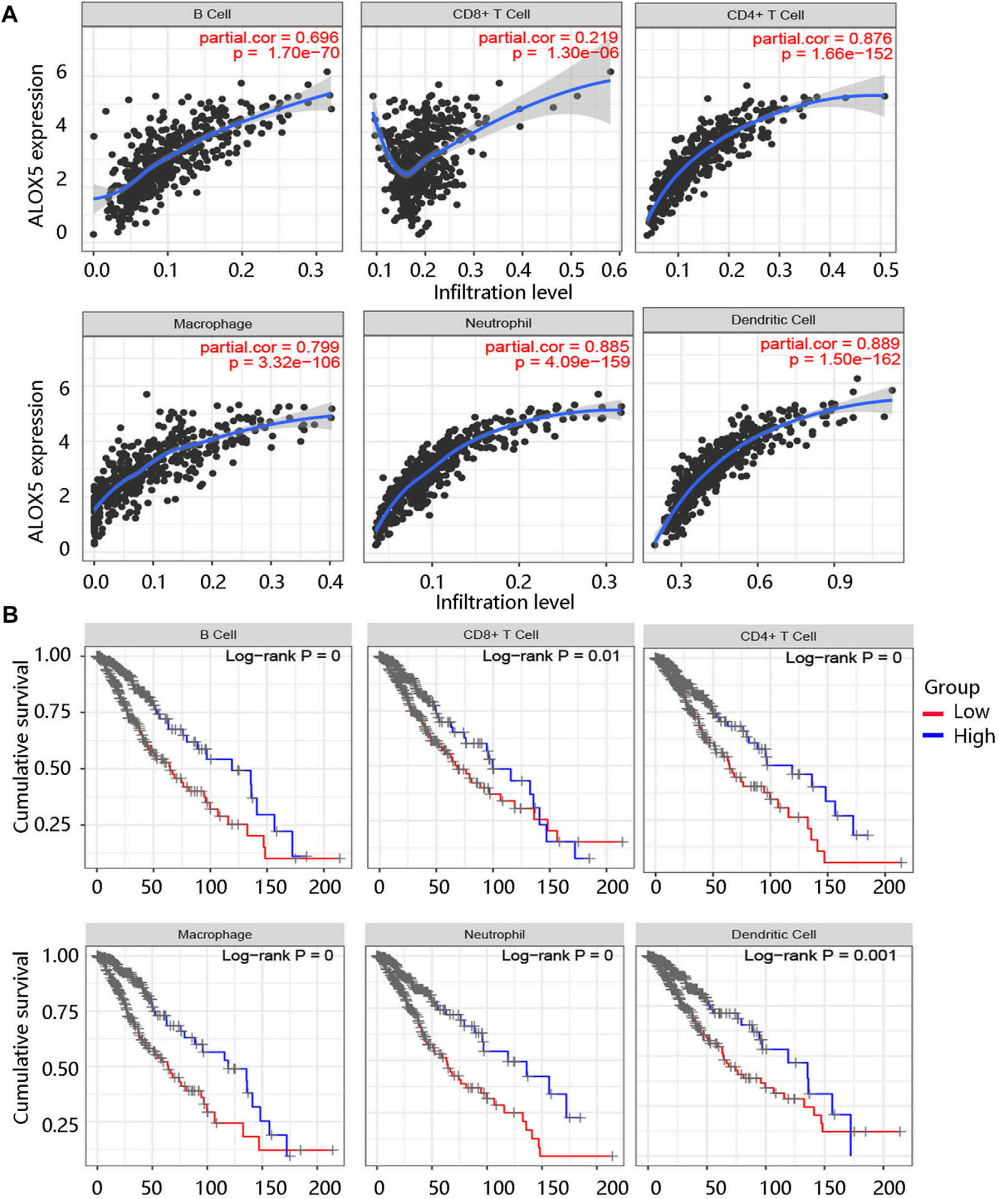
Immune cell infiltration analysis using TIMER algorithm. **(A)** The association of ALOX5 expression with several immune cells including B cell, CD8 + T cell, CD4 + T cell, macrophages, neutrophil, and dendritic cells. **(B)** The survival curves of the different immune cell groups.

The tumor microenvironment (TME) plays an essential role in the progression and pathological features of malignancies (Wang et al., 2022). To explore the relationship between ALOX5 expression and TME in LGG, we assessed the association of ALOX5 expression with Immune score, Stromal score, and Estimate score using the ESTIMATE algorithm. The significant positive associations were illustrated in Figures 8A–C. To exhibit the significance of the TME in LGG, Kaplan-Meier analysis was performed to evaluate the correlation between the Immune score-, Stromal score-, and Estimate score-based groups, and sample survival. The Immune score-high, Stromal score-high, and Estimate score-high groups had decreased survival probability (all *p* < 0.001) (Figures 8D–F). Consistent with this, the ALOX5 mRNA expression was positively related to B cell, CD8 + T cell, CD4 + T cell, macrophage, neutrophil, and dendritic cell (all *p* < 0.001) (Figure 9A). In addition, patients with high immune cell infiltration levels had unfavorable clinical outcomes (Figure 9B). These results suggested that the elevated level of ALOX5 might affect the prognosis of LGG patients partially due to immune infiltration.

### Arachidonate lipoxygenases 5 is involved in immune-related pathways

**FIGURE 10:**
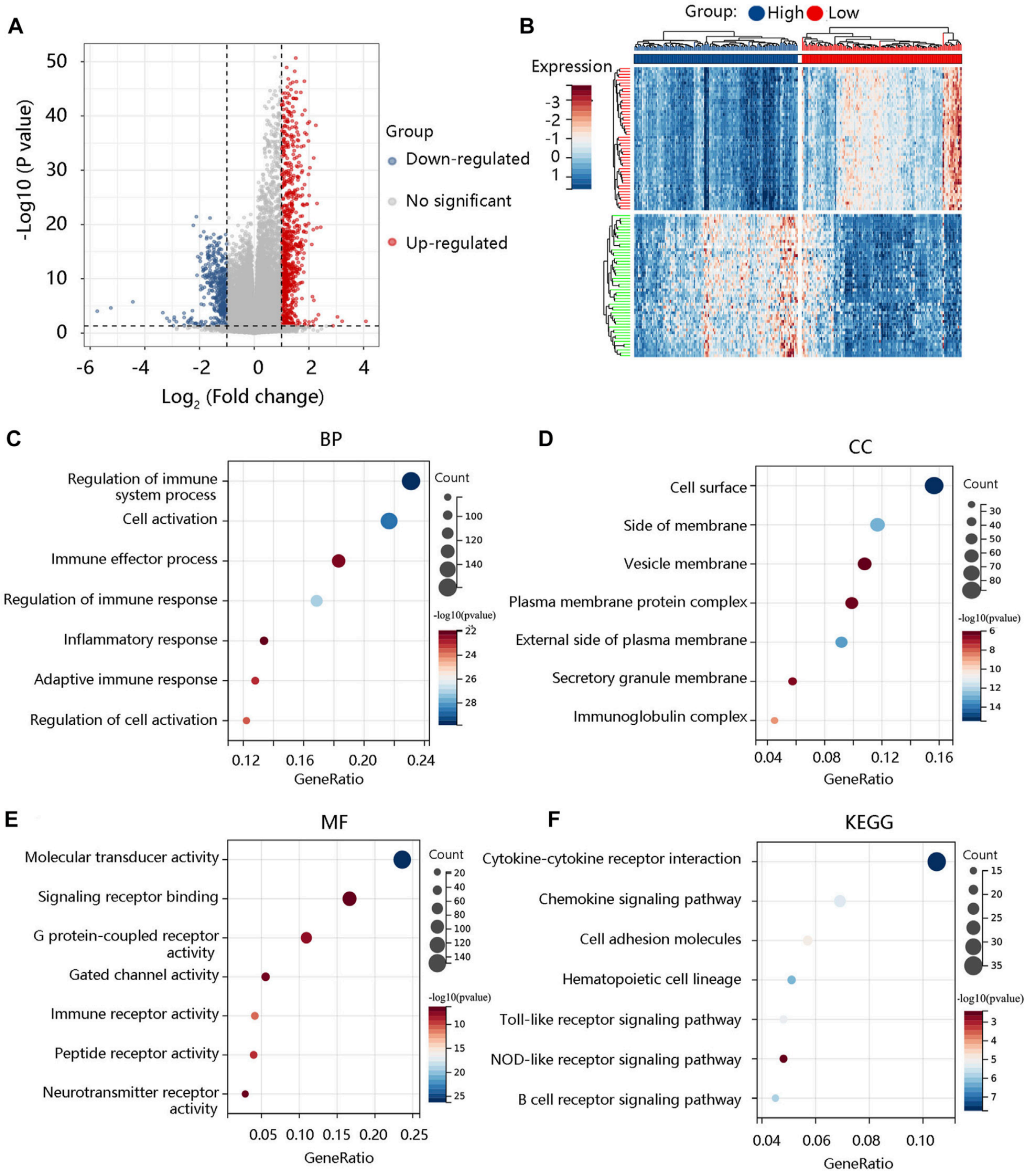
The functional enrichment analysis of the significant differentially expressed genes (DEGs). **(A)** The volcano plot exhibiting all the DEGs between low-grade glioma and normal groups. **(B)** The heat map showing the top 50 DEGs. The **(C)** BP, **(D)** CC, **(E)** MF, and **(F)** KEGG of the significant DEGs. BP, biological process; CC, cellular component; MF, molecular function; KEGG, Kyoto Encyclopedia of Genes and Genomes.

To reveal the pathological role of ALOX5 in LGG, the DEGs between the high- and low- ALOX5 expression groups were identified using the TCGA-LGG data and were shown in the volcano plot (Figure 10A) and the heat map (Figure 10B). As for biological processes, these DEGs were mainly involved in regulation of immune system process, cell activation, and immune effector process (Figure 10C). As for cellular component, they mainly participated in cell surface, side of membrane, and vesicle membrane (Figure 10D). In terms of molecular function, they were mainly involved in molecular transduce activity, signaling receptor binding, and immune receptor activity (Figure 10E). The major KEGG pathways were cytokine-cytokine receptor interaction, cell adhesion molecules, Toll-like receptor signaling pathway, NOD-like receptor signaling pathway, and B cell receptor signaling pathway (Figure 10F).

**FIGURE 11:**
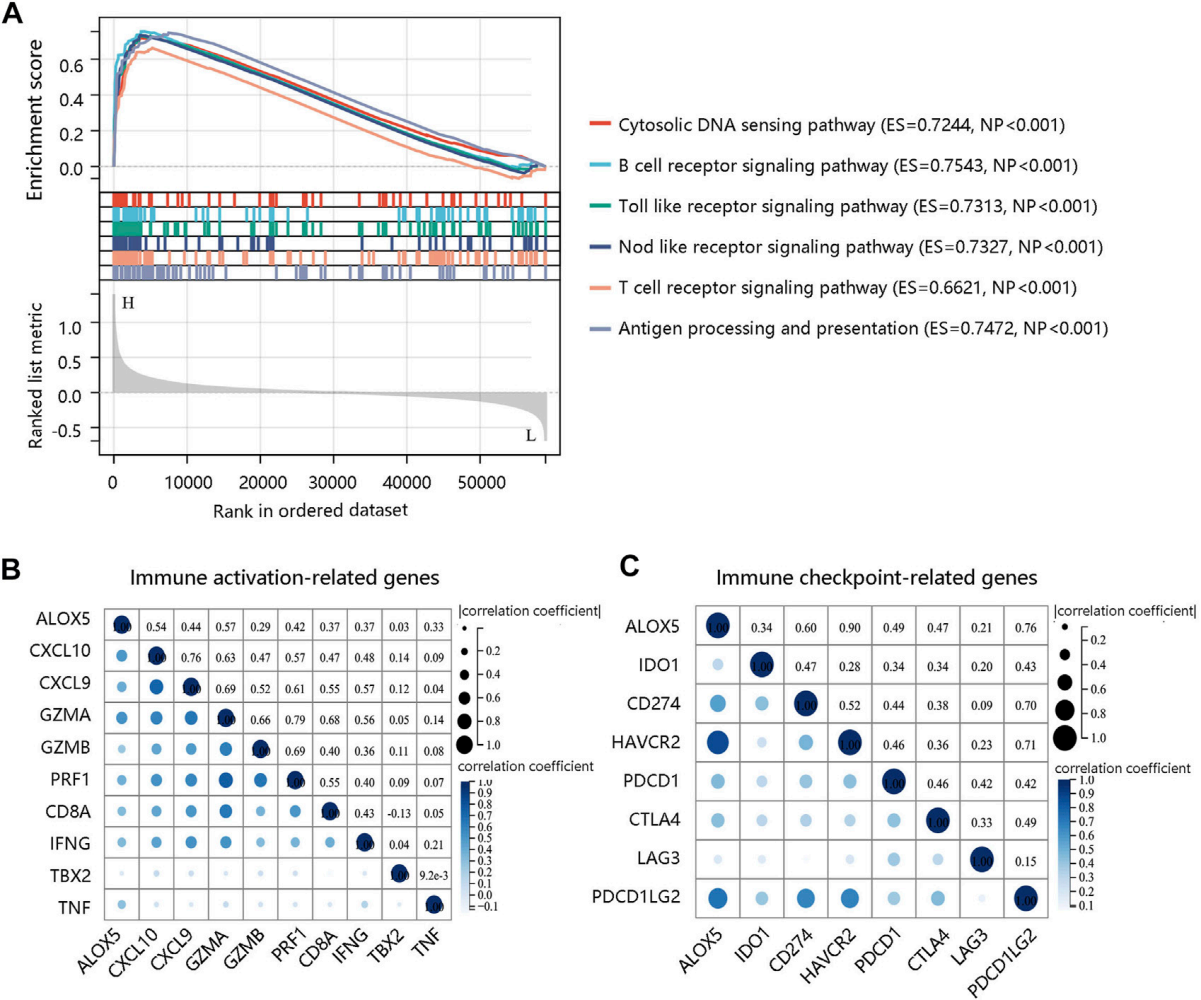
Molecular characteristics of ALOX5. **(A)** GSEA enrichment showing the relative enriched pathways in high ALOX5 expression group. **(B)** Correlations between ALOX5 and immune activation-related genes. **(C)** Correlations between ALOX5 and immune checkpoint-related genes.

To decipher the potential mechanisms of ALOX5, we performed GSEA and found that several immune-related pathways such as B cell receptor signaling pathway, Toll like receptor signaling pathway, Nod like receptor signaling pathway, and T cell receptor signaling pathway were enriched in high ALOX5 expression group (Figure 11A). Therefore, we further explored the relationship between the ALOX5 and immune-related gene sets. ALOX5 was significantly positively correlated not only with immune activation-related genes, but also with immune checkpoint-related genes (Figures 11B,C). These results revealed that ALOX5 might affect the progression of LGG through activating the immune- and inflammation-related pathways.

### Drug sensitivity analysis

**FIGURE 12:**
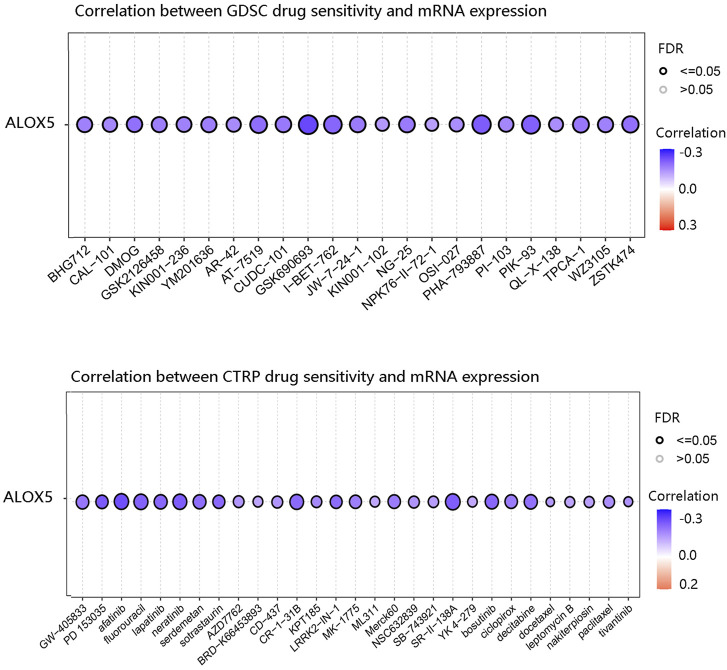
The negative correlation between ALOX5 expression and drug sensitivity based on GDSC and CTRP in the GSCA database.

Genes associated with the sensitivity and resistance of cancer drugs have been extensively studied (Liu et al., 2016; Liu et al., 2020). We predicted the relationship between ALOX5 expression level and drug sensitivity using the GSCA database. ALOX5 was negatively correlated with the sensitivity to various chemotherapeutic drugs such as GSK690693, I-BET-762, PHA-793887, and afatinib (Figure 12).

## Discussion

Herein, we found that the expression of ALOX5 was higher in LGG than that in normal tissue, and its overexpression was closely associated with poor prognosis of patients with LGG. Patients with low methylation levels of ALOX5 had unfavorable clinical outcomes, while those with high immune cell infiltration levels presented shorter survival. The enrichment analysis revealed the possible immune-related pathways regulated by ALOX5. Finally, our result demonstrated that ALOX5 mRNA expression was negatively correlated with drug sensitivity.

**FIGURE 13:**
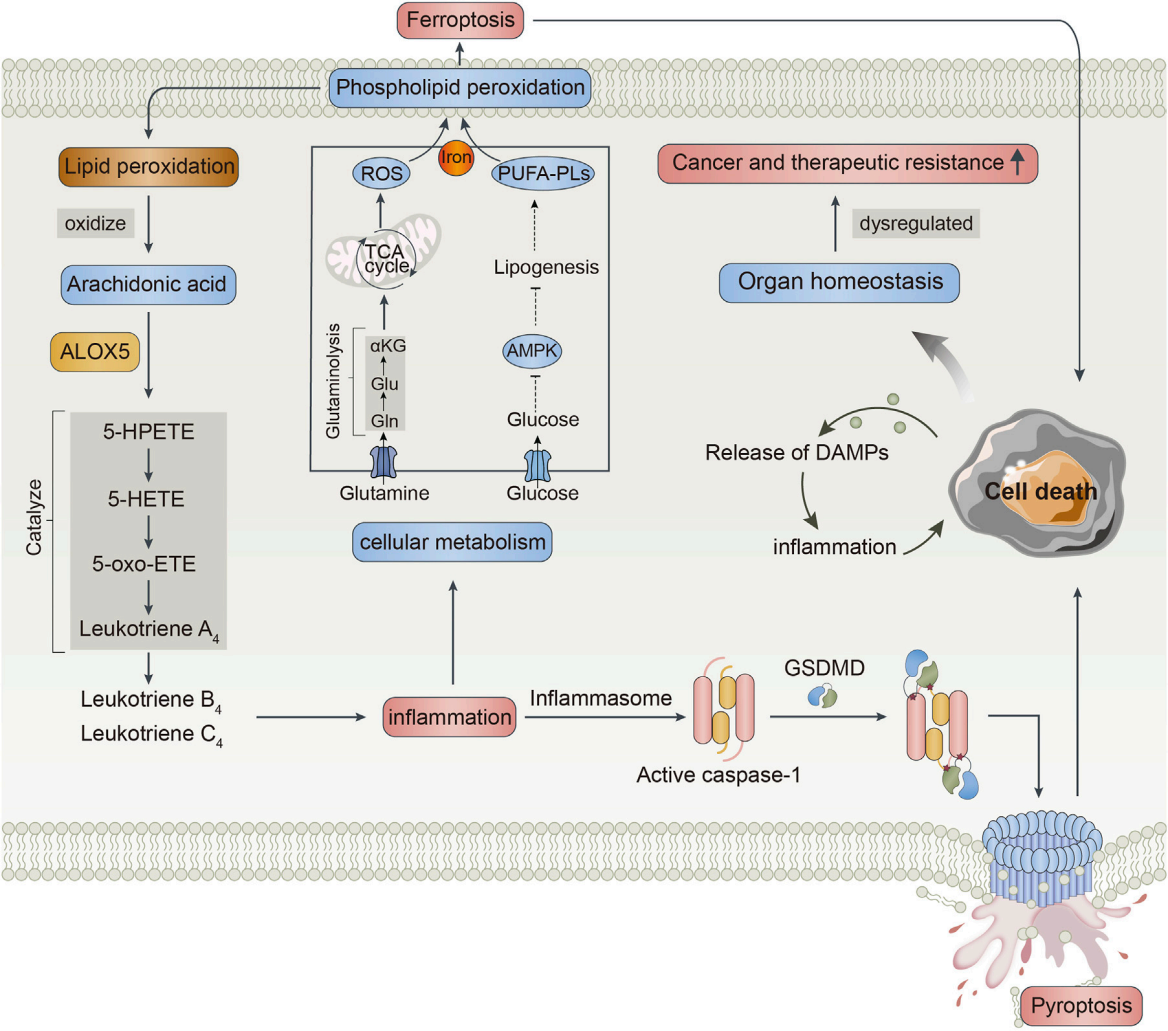
The possible mechanism of ALOX5 involvement in low-grade glioma.

The specific function of ALOX5 is to catalyze AA into 5-HPETE, then into 5-HETE, and further into 5-oxo-eicosatetraenoic acid, which was further catalyzed into LTA4 generating proinflammatory factors including LTB4 and LTC4 by LTA4 hydrolase and LTC4 synthase, respectively (Mouchlis and Dennis, 2019). In addition, ALOX5 is an essential enzyme that mediates lipid peroxidation by producing lipid peroxides (Yang et al., 2022). Excessive lipid peroxidation is easy to occur in phospholipids, contributing to membrane rupture and evoking cell death such as pyroptosis, ferroptosis, and apoptosis (Wiernicki et al., 2020). Pyroptosis is a proinflammatory form of programmed cell death, which is dependent on the activity of caspase acid-specific proteases. In the coupling of the amino-terminal and carboxy-terminal linkers of gasdermin D (GSDMD) by caspases, GSDMD is transferred to the membrane and perforated, causing moisture penetration, cell swelling, and release of inflammatory factors followed by pyroptosis (Chen et al., 2021). Ferroptosis is executed by phospholipid peroxidation which is a process relying on the transition metal iron, phospholipids containing polyunsaturated fatty acid chains (PUFA-PLs), and reactive oxygen species (ROS). Proinflammatory stimuli could alter the cellular metabolism, influencing levels of PUFA-PLs and ROS. Ferroptosis may also have physiological functions in tumor suppression and immune surveillance (Burgos et al., 2020; Jiang X. et al., 2021). The activation of the cell death pathway can also trigger different immune and inflammatory reactions through the release of damage-associated molecular patterns (DAMPs) such as HMGB1, IL-33, IL-18, IL-1α, and IL-1β, further evoking inflammation and cell death (Wojton et al., 2016; Tang D. et al., 2021). Inflammation and cell death can affect each other, regulating organ homeostasis, and once dysregulated, it leads to pathological conditions including cancer and therapeutic resistance (Khan et al., 2020). Therefore, the authors speculated that high ALOX5 expression might affect the poor prognosis of patients with LGG by triggering immune and inflammatory reactions and activating the cell death pathway. On the other hand, LGG patients with high ALOX5 expression might exhibit strong therapeutic resistance, thus having shorter survival. The possible mechanism of ALOX5 involvement in LGG was shown in Figure 13.

Abnormal methylation occupies a crucial role in the induction and progression of cancer including LGG (Mathur et al., 2020; Peng et al., 2021). Hypermethylation of the CpG sites in promoters frequently results in transcriptional silencing, while the hypomethylation of CpG sites in a gene body typically leads to upregulated gene expression (Morgan et al., 2018). This may explain that ALOX5 methylation had a strong negative connection with its mRNA expression. High expression of ALOX5 in LGG tissues might be affected by its low methylation level. Subsequently, we investigated the prognostic significance of ALOX5 DNA methylation and found that hypomethylation of 14 methylated sites in the ALOX5 gene correlated with a worse prognosis. Similarly, the methylation level of GALNT2/14 was negatively correlated with its expression, and patients with GALNT2/14 hypomethylation had shorter OS in lung adenocarcinoma (Yu et al., 2021). Therefore, DNA methylation of ALOX5 might provide additional insight into the treatment and prognosis of LGG.

Increasing evidence proves that tumor-infiltrating immune cells partially influence the prognosis of LGG (Weenink et al., 2019a; Molloy et al., 2020; Wu et al., 2020). Tumor-infiltrating immune cells are part of the complex microenvironment closely related to LGG biological behavior and patient survival (Weenink et al., 2019a; Weenink et al., 2019b). In this analysis, we observed that ALOX5 expression was positively associated with an immune score and several immune cells. Furthermore, high immune infiltration levels lead to a poor prognosis. Therefore, the authors inferred that ALOX5 expression might be implicated in the tumor immune microenvironment, leading to unfavorable clinical outcomes.

Following this, we performed the enrichment analysis to explore the underlying mechanism of ALOX5 in LGG. The results showed that ALOX5 was involved in immune and inflammation-related pathways such as the Toll-like receptor signaling pathway, Nod-like receptor signaling pathway, and antigen processing and presentation. Toll-like receptors are involved in the initiation of the innate and adaptive immune responses and the activation of these receptors triggers an inflammatory response that activates the regulatory pathways of innate and adaptive immunity (Moradi-Marjaneh et al., 2018). Nod-like receptors are also involved in inflammatory responses and tumorigenesis (Liu et al., 2019). Hence, ALOX5 expression might participate in the LGG progression by activation of these pathways. Finally, patients with high ALOX5 expression have higher immune checkpoint molecule expression, indicating that treatment with immune checkpoint inhibitors in the high ALOX5 expression group could attenuate immunosuppression to enhance existing antitumor immunity.

Our study only conducted IHC analysis, but the exploration of potential pathways provided insight into the future experiment. Second, the prognostic role of ALOX5 needs to be validated in prospective cohorts yet may require decades of follow-up.

In conclusion, the high ALOX5 expression serves as an independent prognostic predictor of poor prognosis in LGG. Additionally, ALOX5 expression might be regulated by its methylation level, and ALOX5 expression is also involved in the tumor immune microenvironment. Moreover, ALOX5 expression might be a robust biomarker for improving the prognosis of LGG patients.

## Data Availability

Publicly available datasets were analyzed in this study. This data can be found here: https://www.jianguoyun.com/p/DXtAeYQQp4jxChiLtNMEIAA.
